# Adhesins in *Candida glabrata*

**DOI:** 10.3390/jof4020060

**Published:** 2018-05-20

**Authors:** Bea Timmermans, Alejandro De Las Peñas, Irene Castaño, Patrick Van Dijck

**Affiliations:** 1 KU Leuven, Laboratory of Molecular Cell Biology, Kasteelpark Arenberg 31 bus 2438, 3001 Leuven, Belgium; bea.timmermans@kuleuven.vib.be; 2 VIB-KU Leuven Center for Microbiology, 3001 Leuven, Belgium; 3 IPICYT, División de Biología Molecular, Camino a la Presa San José 2055, C.P., San Luis Potosí 78216 San Luis Potosí, Mexico; cano@ipicyt.edu.mx (A.D.L.P.); icastano@ipicyt.edu.mx (I.C.)

**Keywords:** *Candida glabrata*, adhesin, adhesion, biofilm

## Abstract

The human fungal pathogen *Candida glabrata* is causing more and more problems in hospitals, as this species shows an intrinsic antifungal drug resistance or rapidly becomes resistant when challenged with antifungals. *C. glabrata* only grows in the yeast form, so it is lacking a yeast-to-hyphae switch, which is one of the main virulence factors of *C. albicans*. An important virulence factor of *C. glabrata* is its capacity to strongly adhere to many different substrates. To achieve this, *C. glabrata* expresses a large number of adhesin-encoding genes and genome comparisons with closely related species, including the non-pathogenic *S. cerevisiae*, which revealed a correlation between the number of adhesin-encoding genes and pathogenicity. The adhesins are involved in the first steps during an infection; they are the first point of contact with the host. For several of these adhesins, their importance in adherence to different substrates and subsequent biofilm formation was demonstrated in vitro or in vivo. In this review, we provide an overview of the role of *C. glabrata* adhesins during adhesion and biofilm formation both, under in vitro and in vivo conditions.

## 1. Introduction

*Candida* species pose a major problem in hospitals, as they are the most frequently isolated fungal microorganisms in Healthcare-Associated Infections (HAI) [1,2,3,4]. Major risk factors for *Candida* infections include the use of broad-spectrum antibiotics, immuno-suppression of the host, and the use of medical devices in surgery. While *C. albicans* is still the most common cause of HAI, the isolation rate of non-*C. albicans Candida* species has increased over the years [5]. *C. glabrata* is the second or third most frequently isolated *Candida* species, depending on the geographical area studied [6]. This high incidence can be partially explained by the inherent low susceptibility of *C. glabrata* to the most used class of antifungal drugs, the azoles, and consequently *C. glabrata* HAI are associated with high mortality rates [7].

The use of medical devices, such as catheters, dentures, and prostheses, has increased enormously over the last decades [8,9,10]. These surfaces serve as a substrate for cells to adhere and to form a microbial community called a biofilm. Cells inside a biofilm have an altered gene expression, which gives the biofilm distinct phenotypic properties, e.g., they are frequently highly resistant to antifungal treatment [11], and removal of these medical implants is often necessary to cure the patient, thereby extending the hospital stay and elevating medical costs [9,12]. *Candida* species are also able to form biofilms on medical devices [13]. It was shown that *C. glabrata* forms biofilms on urinary and vascular catheters, prosthetic valves, and pacemakers [9,14,15]. As biofilms are sessile, its formation starts when the cells attach to a surface (adherence), after which the cells divide (proliferation) and form an extracellular matrix (maturation). The cell-surface interaction is mediated by specific proteins in the cell wall, called adhesins, which are widespread across microorganisms [16,17]. A high number of adhesins was predicted to be present in the *C. glabrata* genome, and several were confirmed to be involved in adherence to a specific substrate [18,19]. Furthermore, *C. glabrata* is not polymorphic and only grows as a budding yeast, unlike *C. albicans,* in which the yeast-to-hyphal transition was shown to be one of the most important virulence factors [20]. This indicates that adhesion, and therefore biofilms, are important for virulence in *C. glabrata*. In this review, we will give an update on our current understanding of *C. glabrata* adhesion and its importance in virulence.

## 2. Identification of *C. glabrata* Adhesins from Genomic Data

**Figure 1:**
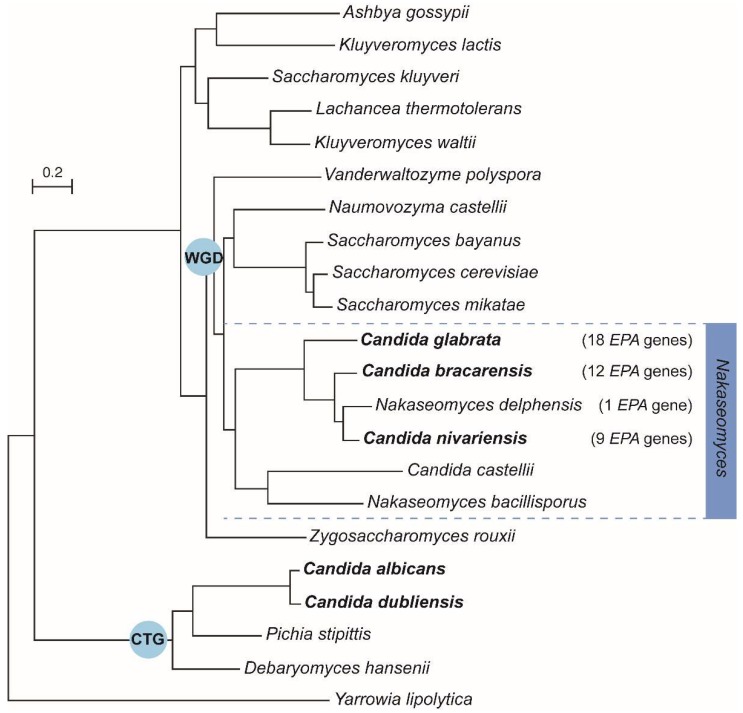
Phylogenetic tree of the subphylum Saccharomycotina, including several *Candida* species and *Saccharomyces cerevisiae*. *C. glabrata* is more closely related to the non-pathogenic *S. cerevisiae* than to the pathogen *C. albicans*, which belongs to the CTG clade. Gabaldon and co-workers found a correlation between the number of *EPA* genes (Epithelial adhesin) in the genome and pathogenicity in the *Nakaseomyces* clade (indicated in the figure). Pathogenic species are depicted in bold (Figure adapted from [28]).

In 2004, the first complete genome sequences of *C. glabrata*, *C. albicans* and several other closely related fungal species were published [21,22,23]. Since then, several studies have been investigating genome evolution by the construction of phylogenetic trees based on different *Candida* species or with a more diverse set of fungal genomes including non-pathogenic species, with the main goal of identifying genes responsible for pathogenesis [23,24,25,26,27]. In these evolutionary trees, the *C. glabrata* genome is always positioned close to the non-pathogenic *S. cerevisiae,* in the clade of species that have undergone a whole genome duplication (WGD), rather than in the CTG clade (translating the CUG codon as serine instead of leucine) containing *C. albicans* and other fungal pathogens (Figure 1) [23,24,25].

A full comparison of the *C. glabrata* and *S. cerevisiae* genomes showed that 337 genes are specific to *C. glabrata* and may therefore provide information about its pathogenicity [29]. Further in depth in silico analysis revealed that a large number of these *C. glabrata* specific genes (51 or 67 genes [18,19]) encode for putative adhesins. 44 out of these 67 adhesin-like encoding genes identified by de Groot et al. [18] are located near the telomeres of all *C. glabrata* chromosomes. Because these subtelomeric regions contain sequence repeats, they are prone to rearrangements or non-allelic homologous recombination, which could explain the expansion of the adhesin-encoding gene families in *C. glabrata*. For some of these adhesins, it has been already shown that their expression is under control of subtelomeric silencing mediated by Rap1, the Sir-complex, and Ku70/80 [30,31,32,33,34]. An update on the effect of chromatin structure and pathways controlling this subtelomeric silencing on adherence in *C. glabrata* can be found in a separate review in this issue by López-Fuentes et al., 2018. Several adhesins also contain internal repetitive sequences (e.g., ‘VSHITT’ tandem repeats in *PWP7* and *AED1* [29,35,36]), which may allow for local chromosomal rearrangements to occur, resulting in different variants of the adhesins. For instance, an increase in the number of the repeats could result in an adhesin that may have its ligand-binding domain projected further out of the cell wall, thereby improving adherence to specific substrates [37,38].

In 2013, new genome sequences from the *Nakaseomyces* clade were published [26]. These species are more closely related to *C. glabrata* than to *S. cerevisiae* (Figure 1), including both pathogenic and non-pathogenic *Candida* and non-*Candida* species. The new genome comparisons of the *Nakaseomyces* species could expose new or confirm already identified factors important for pathogenesis. The latter is true, since Gabaldón and co-workers found a correlation between the number of *EPA* genes (Epithelial adhesin), the largest family of *C. glabrata* adhesins, and pathogenicity in the *Nakaseomyces* clade: the pathogenic *C. bracarensis* and *C. nivariensis* encode for 12 and 9 *EPA* adhesins, respectively, while only one *EPA* adhesin was found in the non-pathogenic *Nakaseomyces delphensis* [26]. This underscores that adhesins are important for the pathogenicity of fungal species.

Recently, *C. glabrata* gene annotation was updated [39], and previously annotated pseudogenes were corrected by splitting the open reading frame (ORF) into two or more new ORFs. Among these, genes were repeatedly predicted as adhesin-like genes, demonstrating that updating and functional analysis of the genome sequence is important. For example, a new gene encoding for a putative GPI-anchored adhesin was identified (*CgNP25*) and probably is part of the *EPA* family [39].

Since sequencing of fungal genomes became affordable, more studies have shown interest in comparing the genomes of clinical isolates with the genome of a reference lab strain [40,41,42,43,44]. All these studies have in common that they show that the *C. glabrata* genome is highly dynamic. This genome plasticity was also found in other pathogens, both prokaryotes and eukaryotes, and allows the pathogen to adapt to environmental changes [45,46,47]. Because *C. glabrata* is considered to be asexual, its genome plasticity is advantageous for its evolution as a pathogen.

Recently, one study sequenced the genomes of two isolates from one patient suffering from oropharyngeal candidiasis [43]. Both clinical isolates had a large number of nucleotide changes compared to the CBS138 reference sequence, and between these two clinical isolates there is a difference of 1024 nucleotides. In depth genome analysis showed that 6 adhesin-like genes of CBS138 were missing in the two clinical isolates and, interestingly, new predicted adhesin-like encoding genes were found exclusively in the clinical isolates. The two clinical isolates contained 101 and 107 adhesin-like genes, which is almost twice the number found in the CBS138 reference strain. Similar to the reference strain, half of the adhesin-like encoding genes were located close to the telomeres [18]. A closer inspection of the predicted adhesin-like encoding genes identified a duplication of several *EPA* genes and also of *PWP* and *AWP* genes [43]. The number of adhesin-like encoding genes present in clinical isolates is significantly higher, which suggests, again, that adhesins play an important role in infection.

In summary, genomic data strongly suggest that the adhesin-like encoding genes present in the *C. glabrata* genome are important for its pathogenicity. As new *Candida* species are identified in clinic, analysing their genomes is extremely relevant and can provide insights into their potential to be a pathogen, as was the case for *C. glabrata*. As a perspective, we think that further genomic analysis of *C. glabrata* clinical isolates and identification of their adhesin-like genes, followed by in-depth expression analysis and functional characterisation, could further increase our knowledge about the mechanism of pathogenesis in *C. glabrata*. 

## 3. Adhesin Families and Ligand Binding Specificity

The *C. glabrata* cell wall structure and composition is largely similar to *S. cerevisiae*, but contains significantly more proteins and mannan, probably due to glycosylation of the cell wall proteins (CWPs). The majority of *C. glabrata* CWPs, among which glycosylphosphatidylinositol (GPI) anchored proteins, is cross-linked to 1,3-β-glucan, while a minority is bound to 1,6-β-glucan via an alkali-sensitive bond [18].

Assuming that adhesins are GPI-anchored proteins, Weig and co-workers used an optimized algorithm on the *C. glabrata* proteome to identify all putative GPI-CWPs [19]. The structural requirements of GPI-CWPs include a N-terminal hydrophobic signal sequence to target the protein to the endoplasmic reticulum, a C-terminal consensus sequence for GPI anchoring and the lack of internal transmembrane domains [48]. In addition, GPI-CWPs have a modular structure with the N-terminal ligand binding domain followed by a low-complexity and usually highly repetitive region with a high percentage of serine and threonine residues that is heavily glycosylated [18]. Out of 106 predicted GPI proteins, 51 were identified as adhesin-like proteins, because they contained adhesin-like structural features [19]; additional putative adhesins were identified by scanning for the conserved ‘VSHITT’ repeat sequences, a typical adhesin structure [18]. Therefore, a total of 67 *C. glabrata* predicted adhesins were identified, and these were classified into several subclasses based on their N-terminal substrate-binding domain [18,19]. The first group includes the Epa (Epithelial **a**dhesion) protein family that contains a ligand-binding domain of approximately 300 amino acids that was identified as the conserved anthrax protective antigen (PA14) domain, suggesting a carbohydrate-binding function [49,50]. This N-terminal ligand binding domain is projected out of the cell through a long and highly glycosylated serine/threonine-rich region. This serine/threonine-rich region is essential for the adherence function of the Epa proteins [37,51]. A second subgroup also has a N-terminal PA14 domain and were therefore named Pwps (PA14 containing wall proteins). The remaining predicted adhesins were organised into five different subgroups, which have more distantly related ligand binding domains [18]. These subgroups are poorly characterized, since most studies have focussed on the Epa family of adhesins. De Groot et al. published that the low-complexity sequence repeats C-terminal of the ligand binding domain, which provides the flexibility to project the substrate-binding domain outside of the cell, is widespread across the different adhesin families [18]. The presence of these adhesins in the *C. glabrata* cell wall was confirmed by the identification of CWPs by mass spectrometry: Awp1, Awp2, Awp3, Awp4, and Epa6 [18], while in another study Epa3, Epa6, Awp2, and Awp4 plus Awp5 and Awp6 (two new adhesin-like cell wall proteins) and several non-unique peptides of other adhesins were identified [52].

The ligand binding specificities of *C. glabrata* adhesins have been determined by modelling of the N-terminal ligand binding domain [51], glycan interaction studies [53,54,55,56], mutagenesis [54], inhibition experiments [57,58], and atomic force microscopy (AFM) studies [59,60]. These studies focused on the Epa family of CWPs, as they were the first *C. glabrata* adhesins characterized (Table 1).

Already in 1999, Cormack and co-workers found that galactose, lactose (galactose β1-4 linked to glucose), and some other glycoconjugates were able to inhibit adhesion of *C. glabrata* to epithelial cells, suggesting a lectin binding function [61]. This theory was supported by the structural analysis of the N-terminal Epa1 binding domain (N-Epa1), co-crystallized with lactose [51]. Later on, a glycan interaction assay using the N-Epa1 showed a strong preference of N-Epa1 to bind glycans containing a terminal galactose residue β1-3 or β1-4 linked to galactose, glucose, N-acetylgalactosamine, or N-acetylglucosamine [53]. N-Epa1 also bound weakly to a terminal galactose α1-3 or α1-4 linked to galactose, N-acetylgalactosamine, or N-acetylglucosamine [53]. The same study investigated the N-terminal ligand binding domains of Epa6 and Epa7, which are highly homologous [62]. It was shown that N-Epa7 has the narrowest ligand specificity, only binding to Galβ1-3Gal or Galβ1-4Glc, while Epa6 does not have any preference and binds to both α- and β-linked glycosides with a terminal galactose residue [53]. These interactions were later confirmed and extended in a study with all 17 Epa proteins of the CBS138 reference strain. Analysis of these 17 proteins resulted in three functional binding classes: class I proteins (Epa1,3,7,9,10) prefer a β1-3 or β1-4 linked galactoside; class II proteins show a preference to β1-3 or β1-6 linked (Epa6,13,22) or sulfated galactosides (Epa12,15,23); and class III proteins prefer to bind acidic sugars (Epa2,19,20,21), sulfated galactosamines (Epa8), or α1-3 linked galactosides (Epa11) [56]. In the human body, these Epa proteins may interact with carbohydrates present in human cells. Indeed, N-Epa1 was found to bind to mucin (the main constituent of mucus and the glycocalyx), fibronectin (major component blood plasma and extracellular matrix), and tumor necrosis factor (TNF-α) on peripheral blood mononuclear cells (PBMCs), and both Epa1 and Epa7 were found to interact with N-glycan structures of kidney and brain tissue [55], while in a competitive binding assay, Epa6 was found to bind to fibronectin [57]. Based on the previous described glycan interaction studies, a lectin-glycan interaction network was constructed that is able to predict a correlation between certain Epas and severe diseases, such as cystic fibrosis or adenocarcinoma [55].

Other studies mutagenized specific residues in the Epa N-terminal domains in order to alter their binding affinities, which gives a good indication of the residues that are involved in substrate binding [53,54,56]. Kuhn and co-workers used inhibition experiments by addition of different carbohydrates to *S. cerevisiae* cells expressing the *C. glabrata* Epa1 adhesin [58]. The results of this study showed that the addition of lactose could only inhibit Epa1-mediated binding to THP-1 cells (monocytes from patient with acute monocytic leukemia), while no effect was seen for other cell lines such as U937 (lymphocytes from histiocytic lymphoma patient) and PBMC (monocytes and lymphocytes of healthy donors). This suggests that the specificity of Epa1 may play a variable role in adhesion or that several adhesions are involved in binding to the substrates tested [58] (Table 1).

**Figure 2:**
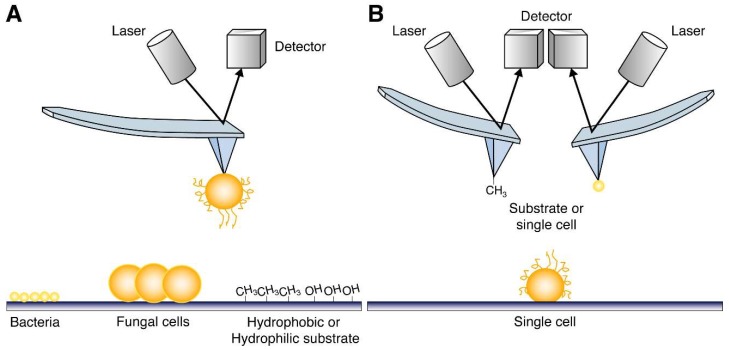
Schematic representation of different AFM strategies used to probe ligand binding specificities of adhesins. (**A**) A single *C. glabrata* cell can be put on the AFM cantilever to probe a certain surface, which can be made of any (hydrophobic or hydrophilic) material or coated with specific biotic substrates, such as bacterial cells or other fungal cells or even other cell types (e.g., human cell lines). (**B**) The cell surface of a single *C. glabrata* cell can be probed in three dimensions using an AFM cantilever tip to which any substrate or other single cell can be attached (Figure based on [60]). Because of all these possibilities to adapt the system, AFM is very attractive to be used in adhesion research.

Atomic force microscopy (AFM) was used to investigate the adhesion profiles of *C. glabrata* cells. Using AFM, adhesion forces of single fungal cells towards different substrates can be measured, providing information about the kind of interaction, e.g., hydrophobic or hydrophilic (Figure 2A) [60]. Using this approach, it was shown that *C. glabrata* single cells have a high adhesive force towards a hydrophobic surface, which was confirmed by probing *C. glabrata* single cells with a CH_3_-bound AFM tip (Figure 2B). In contrast, when an *epa6∆* strain was used in the same experiments, a significantly lower adhesion force and less frequent adhesion events were found, indicating that Epa6 mediates this hydrophobic interaction [59].

## 4. Surface Hydrophobicity and Adherence

*C. glabrata* is able to adhere to a diverse set of surfaces, which can be biotic, as well as abiotic. Adherence to abiotic surfaces is frequently tested in vitro using polystyrene or polypropylene multiwell plates because of the similarity to the Clinical & Laboratory Standards Institute (CLSI) susceptibility tests and because of the simple high throughput screening capacity [70]. Other abiotic surfaces tested include more clinically relevant materials such as polyurethane catheter pieces, denture material, or silicone pieces.

Plastic surfaces, as well as yeast cells, possess a negative surface charge, suggesting a repulsive force [71]. However, *C. glabrata* was found to adhere well to abiotic surfaces. The adhesion force of *C. glabrata* cells is much lower towards a hydrophilic surface compared to a hydrophobic surface [59], and consistent with these results, several studies found a good correlation between an increased adherence capacity of *C. glabrata* cells to abiotic surfaces and a high Cell Surface Hydrophobicity (CSH) [18,63,72]. Thus, adherence to an inert material is mediated by hydrophobic interactions, also called London van der Waals forces [71]. The relative CSH of *C. glabrata* was found to be significantly higher than *C. albicans*, although both show intra-species variation [72,73].

As CSH was found to be correlated to adherence to plastics; one would expect that this interaction is not mediated by specific receptor/adhesin ligand interactions. However, El Kirat-Chatel and co-workers found that adherence to polystyrene was significantly lower in an *epa6∆* strain [59]; the hydrophobic adhesion forces were low in this strain. This indicates that Epa6, which is rich in hydrophobic residues, is, at least partially, responsible for providing the CSH under the conditions tested [59] (Table 1).

## 5. Adherence to Substrates and Biofilm Formation

Adherence is the first step in the formation of a biofilm, which was defined by Donlan and Costerton as a multi-layered structure consisting of a community of microorganisms irreversibly attached to a surface, embedded in an exopolymeric matrix and exhibiting distinctive phenotypic properties [11]. Biofilms are a major concern now in hospitals, as colonization on indwelling medical devices (e.g., urinary catheters) may require removal of the implant, as proper treatment is not available because of different physiology of the biofilm cells compared to planktonic cells, resulting in altered sensitivity towards antimicrobial drugs [1,2,11]. These biofilms can go undetected in the body for years or be life-threatening, depending on the microorganism and host environment [74]. The main problem is that biofilms can serve as a reservoir for seeding infections.

**Figure 3:**
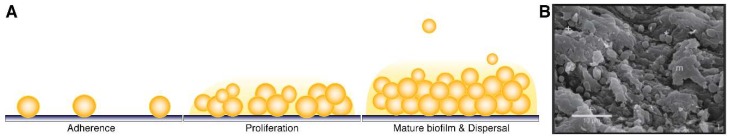
(**A**) Schematic overview of the different stages of biofilm formation in *C. glabrata*. (**B**) Scanning electron microscopy picture of an in vivo mature *C. glabrata* biofilm on a catheter piece, which was recovered from an Intensive Care Unit (ICU) patient. The biofilm is composed of yeast cells (asterix) embedded in extracellular matrix material (m) (Figure from [76]).

A biofilm is formed in different stages [75,76]: First, yeast cells attach to a surface, which can be abiotic or biotic. Second, the adhered cells proliferate on the surface to form microcolonies, after which extracellular matrix is produced. In the final stage, some cells will detach from the biofilm to disperse to other body sites. Because *C. glabrata* only grows by budding, *C. glabrata* mature biofilms are characterized by a dense network of yeast cells embedded in an extracellular matrix (Figure 3), in contrast to *C. albicans,* which forms hyphae during the proliferation phase [69,76,77,78,79]. This is also reflected in the thickness of the biofilms: mature *C. glabrata* biofilms are approximately half as thick (75–90 µm) as *C. albicans* biofilms [69].

Several studies have investigated the expression of *C. glabrata* adhesins, at either transcriptional or protein level. However, it is difficult to compare between studies because of the variation in using different *C. glabrata* strains and the experimental conditions used. For example, the growth medium used to grow the biofilms, or even variations in the pH, changed the expression of adhesins, thus affecting the biofilm morphology [52,80]. Linde et al. even identified that some adhesins, including *EPA3, EPA6,* and *EPA20*, are expressed as different isoforms depending on the growth medium [39].

*EPA1* (Epithelial adhesin 1) was the first *C. glabrata* CWP identified to be important for adhesion in an insertional mutant library screen [61] (Table 1). Epa1 was shown to be largely responsible for in vitro adherence of *C. glabrata* to epithelial cells, since *epa1∆* strains showed a 95 percent reduced adherence, while *S. cerevisiae* becomes highly adherent by heterologous expression of Epa1 [61]. The presence of Ca^2+^ was required for adhesion, and adherence could be inhibited by addition of galactose or lactose (see above). Epa1 is also involved in adherence to human macrophage-like cells: *C. glabrata* or *S. cerevisiae* cells expressing *EPA1* showed a great in vitro adherence to THP-1 cells, and this interaction was inhibited by adding lactose [58]. A similar high adherence was seen with matured human PBMC-derived macrophages, but strikingly, this interaction could not be inhibited by addition of lactose. While the presence of Epa1 was sufficient for binding macrophage-like cells, *C. glabrata* was able to avoid subsequent phagocytosis, whereas *S. cerevisiae* cells expressing Epa1 were phagocytosed by the macrophages after adhesion [58]. Vale-Silva et al. found that the transcription factor Pdr1, known to control the expression of ABC transporters, also regulates *EPA1* expression. Interestingly, several Pdr1 gain of function (GOF) mutants were found to adhere less to THP-1 macrophage-like cells and mouse BMDM macrophages, while adherence to epithelial cells in vitro was increased compared to the wild type strain [64]. A Pdr1 binding site was found in the *EPA1* promoter and the Pdr1^L280F^ GOF mutant leads to *EPA1* overexpression, which was eliminated by deletion of this Pdr1 binding site. Furthermore, introducing Pdr1 GOF mutations did not affect in vitro adherence to epithelial cells in an *epa1∆* strain, indicating that Pdr1 regulates *EPA1* expression [65].

In vivo, there was no difference in virulence between an *epa1∆* or wild type strain in a murine vaginal model and in a gastrointestinal tract infection model [61]. In a murine model of urinary tract infection (UTI), overexpression of *EPA1* leads to significant elevated colonization of bladder and kidneys [65]. Interestingly, deletion of *EPA1* alone caused a small decrease in organ colonization, although not significantly [65]. This is probably due to the involvement of other adhesins, as it was shown by Domergue et al. that the triple mutant strain (*epa1*∆ *epa6*∆ *epa7*∆) showed a significant decreased colonization in the bladder in the murine UTI model [31]. This study also showed that the expression of these adhesins was induced by removing nicotinic acid (NA) from the medium. Consistently, in vitro adherence to uroepithelial cells was significantly lower in presence of excess NA [31].

The insertional mutant library of Cormack and co-workers was used to search for aberrant in vitro biofilm formation in polystyrene multi-well plates. *epa6∆* strains had significant reduced biofilms, while *epa1-5∆* strains were only slightly affected. The expression of *EPA6* and *EPA7* was also shown to be induced in biofilms [67,80]. *EPA6* and *EPA7* have a highly homologous sequence and are both located near the telomeres, where their expression is regulated by subtelomeric silencing [62]. Other mutants that showed poor biofilms were in genes encoding for the chromatin remodelling Swi/Snf complex and a protein kinase *YAK1*. The possible reason for the defect in biofilm formation in strains mutated for these genes is the fact that they are required for the expression of *EPA6* and *EPA7*, possibly by affecting subtelomeric-silencing [67,81]. On the other hand, overproducing biofilm strains were isolated: deletion of *CST6* resulted in a strain with strong biofilm formation capacity and consistently, and the transcription factor Cst6 was shown to be a negative regulator of *EPA6* expression independent of subtelomeric silencing [81]. Another overproducing biofilm strain had an insertion between the two *EPA*-like genes CAGL0I10147g and CAGL0I10200g [81]. Furthermore, the multidrug resistance transporter Tpo1_2 was also found to affect in vitro biofilm formation on polystyrene plates, as its deletion strain showed a 40 percent reduction in biofilm formation. The expression of several adhesins, including *EPA1*, was repressed in the *tpo1_2∆* strain [68].

*C. glabrata* biofilm formation on polystyrene was found to be significantly higher when *C. albicans* was present [66]. In dynamic flow conditions, *C. glabrata* is unable to form a biofilm unless *C. albicans* hyphae are present. Fluorescence microscopy pictures showed that the *C. glabrata* yeast cells were tightly associated along the *C. albicans* hyphae. This association was significantly reduced when the *C. albicans ALS1* and/or *ALS3* genes were deleted. The *C. glabrata* adhesins *EPA8*, *EPA19*, *AWP2*, *AWP7,* and CAGL0F0018g were found to mediate this adherence, and their expression was induced upon incubation with *C. albicans* hyphae [66].

Furthermore, Pwp7 and Aed1 (Adherence to **e**ndothelial **c**ells) were found to play a role in adherence to endothelial cells in vitro, as their deletion strains show a significant reduced adherence compared to the wild type strain [29]. Deletion of *AED2*, located next to *AED1* at the end of chromosome K, did not alter endothelial adherence.

Other studies analyzed the expression of adhesins during biofilm formation. Santos et al. showed that the expression of *EPA1*, *EPA6/7*, *EAP1,* and CAGL0G04125g was upregulated in a 24-h in vitro biofilm compared to 6-h biofilm [68]. Another study compared the adhesin expression of in vitro biofilms to planktonic grown cells: a unique peptide of Awp6 was found exclusively in biofilm cell walls, while peptides of Epa3 were found in the cell wall of biofilm cells and planktonic cells grown in semi-defined yeast growth (SdmYg) medium [52]. At the transcriptional level, the expression of *AWP* adhesins, except *AWP2,* was increased in biofilms compared to planktonic cells. *EPA3* expression was elevated in YPD cultured biofilms, while *EPA7* expression was lower. The expression of *EPA1*, *EPA3*, *EPA7,* and *EPA22* was only induced in SdmYg medium-cultured biofilms [52]. Gómez-Molero et al. compared the CWP profiles of two clinical isolates, which showed an increased in vitro adherence to polystyrene, silicone, and denture material. Mass spectrometry of the isolated CWPs led to the identification of 12 adhesin-like proteins, including Epa3, Epa6, Epa7, and several Awp adhesins, of which 6 were newly identified. Most of the adhesins were shared between the two isolates [63].

Kucharíková and co-workers compared the expression of several adhesins in mature in vitro biofilms formed on polyurethane catheter pieces to mature biofilms isolated from a rat subcutaneous biofilm model. Major differences were found between the in vitro and in vivo biofilms, which was expected, since the growth conditions in vitro can be tightly controlled, while in vivo there is variation originating from the environment (different nutrient states, the state of the immune system, mouse variability, etc.). The expression of *EPA3*, *EPA6*, *AWP2*, *AWP3,* and *AWP5* were significantly higher under the in vivo conditions than the in vitro biofilms, while there was no difference in *EPA1* expression [69].

To determine the expression of specific adhesin-encoding genes under in vivo conditions, Domergue and co-workers made use of recombinant in vivo expression technology (IVET) [31]. *C. glabrata* cells were engineered to become auxotroph for tryptophan and resistant to hygromycin when *EPA6* was expressed. In this way, the percentage of hygromycin-resistant and tryptophan auxotroph colonies can be assessed for the *C. glabrata* recovered from in vivo models of infection. Using IVET, one can determine whether an adhesin was expressed in the tested condition, but it is not possible to address the specific moment of induction or expression. It was shown that *EPA6* was not expressed during in vitro growth or during intravenous infection of mice, while a significant portion of *C. glabrata* cells recovered from an in vivo UTI model was hygromycin-resistant [31]. Using IVET, *EPA2* was found to be expressed in a small but significant part of *C. glabrata* recovered from the liver in a murine model of systemic infection [82].

## 6. Concluding Remarks

Because of its increasing incidence, it is important to investigate the main virulence factors of this pathogen. For many years, it is known that *C. glabrata* encodes for a high number of adhesins, which are considered as one of the main virulence factors of this pathogen. The importance of several *C. glabrata* adhesins in adherence to both biotic and abiotic substrates, as well as for biofilm formation, was demonstrated as presented in this review. Yet, the function for virulence of many other adhesin-encoding genes is still unknown, and, as is clear from recent work, the adhesin-encoding gene family seems to rapidly adapt, as strains with over 100 adhesin genes have now been described [43]. It will be important to continue to study these adhesins in all their aspects: On the one hand, the analysis of newly sequenced genomes of clinical isolates can provide new insights into recent evolutionary events. On the other hand, understanding the environmental conditions that are involved in the regulation of gene expression and posttranscriptional control of specific CWPs could uncover which are the more relevant adhesins to use as targets for antifungal drugs. Yet, it is necessary to complement these expression studies with interaction studies or experiments using mutants to confirm the actual binding of an adhesin to a certain substrate. As several of the *C. glabrata* adhesins are part of protein families, having a similar adhesin structure, redundancy is possible so that the effect of a single deletion can be underestimated due to compensation by other adhesins. With the introduction of CRISPR-Cas9 in *C. glabrata* [83,84], it will be interesting to investigate the functional analysis of a strain containing deletions of several or even all adhesin-encoding genes. This will shed light on their role in virulence in the coming years, as up until now, the in vivo studies published, in which an adhesin deletion mutant was used, did not show differences in virulence.

## Figures and Tables

**Table 1:** Literature overview of expression data (RNA or protein level) of *C. glabrata* adhesins in cells harvested during planktonic growth, during the adhesion phase or during biofilm formation.

Process	Adhesin-Encoding Gene	Substrate	Evidence	Conditions	Read Out	Strains	Reference
Planktonic	*AWP1-4*; *EPA6*		Peptides of Awp1-4 and Epa6 were found in at least one condition tested. Peptides of Awp4 and Epa6 were identified only in stationary-phase cells, while Awp3 was only found in log-phase cells. Awp1 was not identified in the ATCC2001 strain	Cells were grown in YPD or SC medium + 2% glucose and harvested at log phase (OD_600nm_ of 2) or at stationary phase (24 h incubation)	LC-MS/MS on extracted cell walls	ATCC 90876 and ATCC2001	[18]
	*AWP2*; *AWP4*; *EPA3*/*EPA2*; *EPA6*		Awp2, Awp4, Epa6, and Epa3/Epa22 peptide were identified in YPD grown stationary phase cell walls. Awp5 was identified in SdmYg-cultured stationary phase cells	Cells inoculated at OD_600nm_ of 0.1, incubation (16 h, 37 °C, 160 rpm)	LC-MS/MS on extracted cell walls	CBS138	[52]
	*AWP1-7*; *EPA1*; *EPA3*; *EPA6*; *EPA7*; *EPA22*		Relative mRNA expression of clinical isolates to CBS138 reference reveals *EPA1*; *EPA6* and *AWP4* in PEU427 and *EPA6* in PEU382 had an elevated expression. Other adhesins tested showed even lower expression compared to reference strain	Cells from overnight cultures (stationary phase)	RT-PCR from total RNA extraction	CBS138; PEU382; PEU427	[63]
Adhesion	*EPA1*	Epithelial cells	*epa1∆* shows 95% reduced adhesion compared to wild type	Exponential phase cells in RPMI medium, added to Lec2 or HEp2 cells, briefly centrifugated (1–2 min, 500 g), 60 min incubation	Colony Forming Units (CFU)	BG2; *epa1∆*;BY4741; BY4741 + p*EPA1*	[61]
			99% of *S. cerevisiae* adhere to Lec2 cells by heterologous expression of Epa1				
	*EPA1*	Macrophage-like cells	*epa1∆* shows reduced adherence to THP-1 or PBMC cells	Cells in HBSS medium were added (MOI 3:1) to mammalian macrophage-like cells (10^6^ cells/mL) in 96-well plates, 45 min, 37 °C, 5% CO_2_	Fluorescent emission or flow cytometry	BG2; *epa1∆*; S288C; S288C + p*EPA1*	[58]
			*S. cerevisiae* adheres to THP-1 or PBMC cells by heterologous expression of Epa1				
		Macrophage-like cells	Strains expressing *PDR1*^L280F^ hyperactive allele adhere less to PBMCs	*C. glabrata* cells were added to THP-1 cells treated with cytochalasin D (inhibition of phagocytosis), incubated (30 min, 37 °C, 5% CO_2_)	Colony Forming Units (CFU)	DSY562 (*PDR1*^WT^); DSY562 (*PDR1*^L280F^); DSY565 (*PDR1*^L280F^)	[64]
	*EPA1*	Epithelial cells	*PDR1*^L280F^ strains show increased adherence to epithelial cells, concomitant with elevated *EPA1* expression	*C. glabrata* cells were added to Lec2, HeLa or Caco-2 cell lines, centrifuated (1 min, 200 g), incubated (30 min, 37 °C, 5% CO_2_)	Colony Forming Units (CFU)	CBS138; BG2; *epa1∆*; DSY562; DSY565 with either *PDR1*^WT^ or *PDR1*^L280F^	[64,65]
	*EPA6*	Polystyrene (96-well plate)	*epa6∆* shows significant lower in vitro adhesion	10^6^ cells/mL in RPMI 1640 (pH 7.0), 90 min, 37 °C, static;	XTT formazan production	ATCC2001; *epa6∆*	[59]
		Hydrophobic groups	*epa6∆* has smaller adhesion forces and shorter ruptures to CH_3_ than wild type	Probing of single *C. glabrata* cells with hydrophobic group (CH_3_)	AFM interaction forces		
	*AED1; PWP7*	Endothelial cells	*pwp7∆* and *aed1∆* strains had 66% and 50% reduced adhesion respectively, while *aed2∆* strain showed wild type adherence	*C. glabrata* was added to human umbilical vein endothelial cells and incubated (15–60 min, 37 °C)	Colony Forming Units (CFU)	BG14; *pwp7∆*; *aed1∆*; *aed2∆*	[29]
		*C. albicans* hyphae	*EPA8, EPA19, AWP2,* AWP7 and CAGL0F0018g expression were induced upon incubation with *C. albicans* hyphae	*C. albicans* germinated or yeast cells and *C. glabrata* (1:1 ratio), incubated (60 min)	Scanning electron microscopy	BG2; DSY562; VSY55	[66]
Biofilm	*EPA6*	Polystyrene (96-well plate)	*epa6∆* shows 30% reduced biofilm formation, while superbiofilms were formed when *EPA6* was overexpressed	Cells in SC medium, overnight incubation at 37 °C	XTT formazan production	BG2; *epa6∆*	[67]
	*EPA6*	Polystyrene (96-well plate)	*epa6∆* shows significant lower in vitro biofilm formation	After adherence, washed cells were submerged in fresh RPMI 1640 medium, 24 h, 37 °C	XTT formazan production	ATCC2001; *epa6∆*	[59]
	*EPA1*, *EPA6/7*, *EAP1* and CAGL0G04125g	Polystyrene (petri plate)	*EPA1*, *EPA6/7*, *EAP1* and CAGL0G04125g expression was upregulated in 24 h biofilms compared to 6 h biofilms	cells in RPMI 1640 (pH 4) at OD_600nm_ of 0.05, incubation (6 or 24 h, 30 °C, 30 rpm)	RT-PCR from total RNA extraction	CBS138	[68]
	*AWP6*	Polystyrene (petri plate)	Awp6 peptides were identified only in biofilm cell walls Epa3 peptides were found in both planktonic and SdmYg biofilms	Biofilms: Cells at OD_600nm_ of 0.2, incubation (24 h, 37 °C) Planktonic: Cells at OD_600nm_ of 0.1, incubation (16 h, 37 °C, 160 rpm)	LC-MS/MS on extracted cell walls	CBS138	[52]
	*AWP1-7; EPA1, EPA3, EPA6, EPA7, EPA22*		*AWP1, AWP3, AWP5, AWP7, EPA3* expression upregulated in biofilms (YPD and SdmYg medium); *AWP4* and *AWP6* expression upregulated, *AWP2* and *EPA7*downregulated in YPD biofilms; *EPA1* and *EPA22* expression upregulated in SdmYg biofilms	Biofilms: Cells at OD_600nm_ of 0.2, incubation (24 h, 37 °C) Planktonic: Cells at OD_600nm_ of 0.1, incubation (37 °C, 160 rpm) to 0D_600nm_ = 1.0	RT-PCR from total RNA extraction		
	*EC21:I21PA3-7; AWP2; AWP4; AWP6; AWP8-13EPA3-7; AWP2; AWP4; AWP6; AWP8-13*	Polystyrene (petri plate)	Peptides of Epa3, Epa6, Awp2, Awp4, Awp6, and Awp12 were identified in the CBS138 strain. Epa3, Epa6, Epa7, Awp2, Awp4, Awp6, Awp8 were identified in both PEU382 and PEU427 clinical isolates. Awp9, Awp10, Awp12, and Awp13, were unique to PEU427 while Awp11 was only found in PEU382. Epa4 and Epa5 peptides were found in PEU427, while absent in in CBS138 strain.	Cells inoculated in YPD medium, incubation (37 °C) to logarithmic phase and seeded in Petri dishes, incubation (24 h, 37 °C)	LC-MS/MS on extracted cell walls	CBS138; PEU382; PEU427	[63]
	*EPA1; EPA3; EPA6; AWP1-7*	Polyurethane (catheter)	Expression of *EPA3, EPA6, AWP2, AWP3,* and *AWP5* was significantly higher in in vivo biofilms compared to in vitro biofilms	In vitro biofilms: Cells were added to catheters in RPMI 1640 medium and grown for 6 days in vivo biofilms. Cells were added to catheters in RPMI 1640 medium, and, after the period of adhesion (90 min, 37 °C), the catheters were washed and implanted in the back of the animals for 6 days	RT-PCR from total RNA extraction	ATCC2001	[69]

## References

[1] European Centre for Disease Prevention and Control (2016). Annual Epidemiological Report 2016—Surgical Site Infections.

[2] European Centre for Disease Prevention and Control Healthcare-Associated Infections Acquired in Intensive Care Units. https://ecdc.europa.eu/en/publications-data/healthcare-associated-infections-acquired-intensive-care-units-annual.

[3] Zarb P., Coignard B., Griskeviciene J., Muller A., Vankerckhoven V., Weist K., Goossens M., Vaerenberg S., Hopkins S., Catry B. (2012). The European Centre for Disease Prevention and Control (ECDC) pilot point prevalence survey of healthcare-associated infections and antimicrobial use. Eurosurveillance.

[4] Pfaller M.A., Diekema D.J. (2007). Epidemiology of invasive candidiasis: A persistent public health problem. Clin. Microbiol. Rev..

[5] Pfaller M.A., Diekema D.J., Gibbs D.L., Newell V.A., Ellis D., Tullio V., Rodloff A., Fu W., Ling T.A. (2010). Global Antifungal Surveillance Group. Results from the ARTEMIS DISK Global Antifungal Surveillance Study, 1997 to 2007: A 10.5-year analysis of susceptibilities of *Candida* Species to fluconazole and voriconazole as determined by CLSI standardized disk diffusion. J. Clin. Microbiol..

[6] Guinea J. (2014). Global trends in the distribution of *Candida* species causing candidemia. Clin. Microbiol. Infect..

[7] Choi H.K., Jeong S.J., Lee H.S., Chin B.S., Choi S.H., Han S.H., Kim M.S., Kim C.O., Choi J.Y., Song Y.G. (2009). Blood stream infections by *Candida glabrata* and *Candida krusei*: A single-center experience. Korean J. Intern. Med..

[8] Von Eiff C., Jansen B., Kohnen W., Becker K. (2005). Infections associated with medical devices: Pathogenesis, management and prophylaxis. Drugs.

[9] Kojic E.M., Darouiche R.O. (2004). *Candida* infections of medical devices. Clin. Microbiol. Rev..

[10] Hawser S.P., Douglas L.J. (1994). Biofilm formation by *Candida* species on the surface of catheter materials in vitro. Infect. Immun..

[11] Donlan R.M., Costerton J.W. (2002). Biofilms: Survival mechanisms of clinically relevant microorganisms. Clin. Microbiol. Rev..

[12] Darouiche R.O. (2004). Treatment of infections associated with surgical implants. N. Engl. J. Med..

[13] Kumamoto C.A. (2002). *Candida* biofilms. Curr. Opin. Microbiol..

[14] Richards M.J., Edwards J.R., Culver D.H., Gaynes R.P. (2000). Nosocomial infections in combined medical-surgical intensive care units in the United States. Infect. Control Hosp. Epidemiol..

[15] Coco B.J., Bagg J., Cross L.J., Jose A., Cross J., Ramage G. (2008). Mixed *Candida albicans* and *Candida glabrata* populations associated with the pathogenesis of denture stomatitis. Oral. Microbiol. Immunol..

[16] Kline K.A., Falker S., Dahlberg S., Normark S., Henriques-Normark B. (2009). Bacterial adhesins in host-microbe interactions. Cell Host Microbe.

[17] de Groot P.W., Bader O., de Boer A.D., Weig M., Chauhan N. (2013). Adhesins in human fungal pathogens: Glue with plenty of stick. Eukaryot. Cell.

[18] de Groot P.W., Kraneveld E.A., Yin Q.Y., Dekker H.L., Gross U., Crielaard W., de Koster C.G., Bader O., Klis F.M., Weig M. (2008). The cell wall of the human pathogen *Candida glabrata*: Differential incorporation of novel adhesin-like wall proteins. Eukaryot. Cell.

[19] Weig M., Jansch L., Gross U., De Koster C.G., Klis F.M., De Groot P.W. (2004). Systematic identification in silico of covalently bound cell wall proteins and analysis of protein-polysaccharide linkages of the human pathogen *Candida glabrata*. Microbiology.

[20] Biswas S., Van Dijck P., Datta A. (2007). Environmental sensing and signal transduction pathways regulating morphopathogenic determinants of *Candida albicans*. Microbiol. Mol. Biol. Rev..

[21] Dujon B., Sherman D., Fischer G., Durrens P., Casaregola S., Lafontaine I., De Montigny J., Marck C., Neuveglise C., Talla E. (2004). Genome evolution in yeasts. Nature.

[22] Jones T., Federspiel N.A., Chibana H., Dungan J., Kalman S., Magee B.B., Newport G., Thorstenson Y.R., Agabian N., Magee P.T. (2004). The diploid genome sequence of *Candida albicans*. Proc. Natl. Acad. Sci. USA.

[23] Butler G., Rasmussen M.D., Lin M.F., Santos M.A., Sakthikumar S., Munro C.A., Rheinbay E., Grabherr M., Forche A., Reedy J.L. (2009). Evolution of pathogenicity and sexual reproduction in eight *Candida* genomes. Nature.

[24] Fitzpatrick D.A., Logue M.E., Stajich J.E., Butler G. (2006). A fungal phylogeny based on 42 complete genomes derived from supertree and combined gene analysis. BMC Evol. Biol..

[25] Marcet-Houben M., Gabaldon T. (2009). The tree versus the forest: The fungal tree of life and the topological diversity within the yeast phylome. PLoS ONE.

[26] Gabaldon T., Martin T., Marcet-Houben M., Durrens P., Bolotin-Fukuhara M., Lespinet O., Arnaise S., Boisnard S., Aguileta G., Atanasova R. (2013). Comparative genomics of emerging pathogens in the *Candida glabrata* clade. BMC Genom..

[27] Gabaldon T., Naranjo-Ortiz M.A., Marcet-Houben M. (2016). Evolutionary genomics of yeast pathogens in the Saccharomycotina. FEMS Yeast Res..

[28] Gabaldon T., Carrete L. (2016). The birth of a deadly yeast: Tracing the evolutionary emergence of virulence traits in *Candida glabrata*. FEMS Yeast Res..

[29] Desai C., Mavrianos J., Chauhan N. (2011). *Candida glabrata* Pwp7p and Aed1p are required for adherence to human endothelial cells. FEMS Yeast Res..

[30] De Las Peñas A., Pan S.J., Castaño I., Alder J., Cregg R., Cormack B.P. (2003). Virulence-related surface glycoproteins in the yeast pathogen *Candida glabrata* are encoded in subtelomeric clusters and subject to *RAP1*- and *SIR*-dependent transcriptional silencing. Genes Dev..

[31] Domergue R., Castaño I., De Las Peñas A., Zupancic M., Lockatell V., Hebel J.R., Johnson D., Cormack B.P. (2005). Nicotinic acid limitation regulates silencing of *Candida* adhesins during UTI. Science.

[32] Gallegos-Garcia V., Pan S.J., Juarez-Cepeda J., Ramirez-Zavaleta C.Y., Martin-del-Campo M.B., Martinez-Jimenez V., Castaño I., Cormack B., De Las Peñas A. (2012). A novel downstream regulatory element cooperates with the silencing machinery to repress *EPA1* expression in *Candida glabrata*. Genetics.

[33] Halliwell S.C., Smith M.C., Muston P., Holland S.L., Avery S.V. (2012). Heterogeneous expression of the virulence-related adhesin Epa1 between individual cells and strains of the pathogen *Candida glabrata*. Eukaryot. Cell.

[34] Juarez-Reyes A., De Las Peñas A., Castaño I. (2011). Analysis of subtelomeric silencing in *Candida glabrata*. Methods Mol. Biol..

[35] Thierry A., Bouchier C., Dujon B., Richard G.F. (2008). Megasatellites: A peculiar class of giant minisatellites in genes involved in cell adhesion and pathogenicity in *Candida glabrata*. Nucleic Acids Res..

[36] Thierry A., Dujon B., Richard G.F. (2010). Megasatellites: A new class of large tandem repeats discovered in the pathogenic yeast *Candida glabrata*. Cell. Mol. Life Sci..

[37] Frieman M.B., McCaffery J.M., Cormack B.P. (2002). Modular domain structure in the *Candida glabrata* adhesin Epa1p, a beta1,6 glucan-cross-linked cell wall protein. Mol. Microbiol..

[38] Verstrepen K.J., Jansen A., Lewitter F., Fink G.R. (2005). Intragenic tandem repeats generate functional variability. Nat. Genet..

[39] Linde J., Duggan S., Weber M., Horn F., Sieber P., Hellwig D., Riege K., Marz M., Martin R., Guthke R. (2015). Defining the transcriptomic landscape of *Candida glabrata* by RNA-Seq. Nucleic Acids Res..

[40] Polakova S., Blume C., Zarate J.A., Mentel M., Jorck-Ramberg D., Stenderup J., Piskur J. (2009). Formation of new chromosomes as a virulence mechanism in yeast *Candida glabrata*. Proc. Natl. Acad. Sci. USA.

[41] Muller H., Thierry A., Coppee J.Y., Gouyette C., Hennequin C., Sismeiro O., Talla E., Dujon B., Fairhead C. (2009). Genomic polymorphism in the population of *Candida glabrata*: Gene copy-number variation and chromosomal translocations. Fungal Genet. Biol..

[42] Ahmad K.M., Ishchuk O.P., Hellborg L., Jorgensen G., Skvarc M., Stenderup J., Jorck-Ramberg D., Polakova S., Piskur J. (2013). Small chromosomes among Danish *Candida glabrata* isolates originated through different mechanisms. Anton. Leeuwenhoek J. Microbiol..

[43] Vale-Silva L., Beaudoing E., Tran V.D.T., Sanglard D. (2017). Comparative Genomics of Two Sequential *Candida glabrata* Clinical Isolates. Genes Genomes Genet..

[44] Bader O., Schwarz A., Kraneveld E.A., Tangwattanachuleeporn M., Schmidt P., Jacobsen M.D., Gross U., De Groot P.W., Weig M. (2012). Gross karyotypic and phenotypic alterations among different progenies of the *Candida glabrata* CBS138/ATCC2001 reference strain. PLoS ONE.

[45] Thieme F., Koebnik R., Bekel T., Berger C., Boch J., Buttner D., Caldana C., Gaigalat L., Goesmann A., Kay S. (2005). Insights into genome plasticity and pathogenicity of the plant pathogenic bacterium *Xanthomonas campestris* pv. *vesicatoria* revealed by the complete genome sequence. J. Bacteriol..

[46] Selmecki A., Forche A., Berman J. (2010). Genomic plasticity of the human fungal pathogen *Candida albicans*. Eukaryot. Cell.

[47] Dobrindt U., Hacker J. (2001). Whole genome plasticity in pathogenic bacteria. Curr. Opin. Microbiol..

[48] De Groot P.W., Hellingwerf K.J., Klis F.M. (2003). Genome-wide identification of fungal GPI proteins. Yeast.

[49] Rigden D.J., Mello L.V., Galperin M.Y. (2004). The PA14 domain, a conserved all-beta domain in bacterial toxins, enzymes, adhesins and signaling molecules. Trends Biochem. Sci..

[50] Maestre-Reyna M., Diderrich R., Veelders M.S., Eulenburg G., Kalugin V., Bruckner S., Keller P., Rupp S., Mosch H.U., Essen L.O. (2012). Structural basis for promiscuity and specificity during *Candida glabrata* invasion of host epithelia. Proc. Natl. Acad. Sci. USA.

[51] Ielasi F.S., Decanniere K., Willaert R.G. (2012). The epithelial adhesin 1 (Epa1p) from the human-pathogenic yeast *Candida glabrata*: Structural and functional study of the carbohydrate-binding domain. Acta Crystallogr. Sect. D.

[52] Kraneveld E.A., de Soet J.J., Deng D.M., Dekker H.L., de Koster C.G., Klis F.M., Crielaard W., de Groot P.W. (2011). Identification and differential gene expression of adhesin-like wall proteins in *Candida glabrata* biofilms. Mycopathologia.

[53] Zupancic M.L., Frieman M., Smith D., Alvarez R.A., Cummings R.D., Cormack B.P. (2008). Glycan microarray analysis of *Candida glabrata* adhesin ligand specificity. Mol. Microbiol..

[54] Ielasi F.S., Verhaeghe T., Desmet T., Willaert R.G. (2014). Engineering the carbohydrate-binding site of Epa1p from *Candida glabrata*: Generation of adhesin mutants with different carbohydrate specificity. Glycobiology.

[55] Ielasi F.S., Alioscha-Perez M., Donohue D., Claes S., Sahli H., Schols D., Willaert R.G. (2016). Lectin-Glycan Interaction Network-Based Identification of Host Receptors of Microbial Pathogenic Adhesins. MBio.

[56] Diderrich R., Kock M., Maestre-Reyna M., Keller P., Steuber H., Rupp S., Essen L.O., Mosch H.U. (2015). Structural Hot Spots Determine Functional Diversity of the *Candida glabrata* Epithelial Adhesin Family. J. Biol. Chem..

[57] Zajac D., Karkowska-Kuleta J., Bochenska O., Rapala-Kozik M., Kozik A. (2016). Interaction of human fibronectin with *Candida glabrata* epithelial adhesin 6 (Epa6). Acta Biochim. Pol..

[58] Kuhn D.M., Vyas V.K. (2012). The *Candida glabrata* adhesin Epa1p causes adhesion, phagocytosis, and cytokine secretion by innate immune cells. FEMS Yeast Res..

[59] El-Kirat-Chatel S., Beaussart A., Derclaye S., Alsteens D., Kucharikova S., Van Dijck P., Dufrene Y.F. (2015). Force nanoscopy of hydrophobic interactions in the fungal pathogen *Candida glabrata*. ACS Nano.

[60] El-Kirat-Chatel S., Beaussart A., Boyd C.D., O’Toole G.A., Dufrene Y.F. (2014). Single-cell and single-molecule analysis deciphers the localization, adhesion, and mechanics of the biofilm adhesin LapA. ACS Chem. Biol..

[61] Cormack B.P., Ghori N., Falkow S. (1999). An adhesin of the yeast pathogen *Candida glabrata* mediating adherence to human epithelial cells. Science.

[62] Castaño I., Pan S.J., Zupancic M., Hennequin C., Dujon B., Cormack B.P. (2005). Telomere length control and transcriptional regulation of subtelomeric adhesins in *Candida glabrata*. Mol. Microbiol..

[63] Gomez-Molero E., de Boer A.D., Dekker H.L., Moreno-Martinez A., Kraneveld E.A., Chauhan N., Weig M., de Soet J.J., de Koster C.G. (2015). Proteomic analysis of hyperadhesive *Candida glabrata* clinical isolates reveals a core wall proteome and differential incorporation of adhesins. FEMS Yeast Res..

[64] Vale-Silva L., Ischer F., Leibundgut-Landmann S., Sanglard D. (2013). Gain-of-function mutations in *PDR1*, a regulator of antifungal drug resistance in *Candida glabrata*, control adherence to host cells. Infect. Immun..

[65] Vale-Silva L.A., Moeckli B., Torelli R., Posteraro B., Sanguinetti M., Sanglard D. (2016). Upregulation of the Adhesin Gene *EPA1* Mediated by *PDR1* in *Candida glabrata* Leads to Enhanced Host Colonization. mSphere.

[66] Tati S., Davidow P., McCall A., Hwang-Wong E., Rojas I.G., Cormack B., Edgerton M. (2016). *Candida glabrata* Binding to *Candida albicans* Hyphae Enables Its Development in Oropharyngeal Candidiasis. PLoS Pathog..

[67] Iraqui I., Garcia-Sanchez S., Aubert S., Dromer F., Ghigo J.M., d’enfert C., Janbon G. (2005). The Yak1p kinase controls expression of adhesins and biofilm formation in *Candida glabrata* in a Sir4p-dependent pathway. Mol. Microbiol..

[68] Santos R., Costa C., Mil-Homens D., Romao D., de Carvalho C.C., Pais P., Mira N.P., Fialho A.M., Teixeira M.C. (2017). The multidrug resistance transporters CgTpo1_1 and CgTpo1_2 play a role in virulence and biofilm formation in the human pathogen *Candida glabrata*. Cell. Microbiol..

[69] Kucharikova S., Neirinck B., Sharma N., Vleugels J., Lagrou K., Van Dijck P. (2015). In vivo *Candida glabrata* biofilm development on foreign bodies in a rat subcutaneous model. J. Antimicrob. Chemother..

[70] Krom B.P., Willems H.M. (2016). In Vitro Models for Candida Biofilm Development.

[71] Klotz S.A., Drutz D.J., Zajic J.E. (1985). Factors governing adherence of *Candida* species to plastic surfaces. Infect. Immun..

[72] Luo G., Samaranayake L.P. (2002). *Candida glabrata*, an emerging fungal pathogen, exhibits superior relative cell surface hydrophobicity and adhesion to denture acrylic surfaces compared with *Candida albicans*. APMIS.

[73] Hazen K.C., Plotkin B.J., Klimas D.M. (1986). Influence of growth conditions on cell surface hydrophobicity of *Candida albicans* and *Candida glabrata*. Infect. Immun..

[74] Vertes A., Hitchins V., Phillips K.S. (2012). Analytical challenges of microbial biofilms on medical devices. Anal. Chem..

[75] Nobile C.J., Fox E.P., Nett J.E., Sorrells T.R., Mitrovich Q.M., Hernday A.D., Tuch B.B., Andes D.R., Johnson A.D. (2012). A Recently Evolved Transcriptional Network Controls Biofilm Development in *Candida albicans*. Cell.

[76] Paulitsch A.H., Willinger B., Zsalatz B., Stabentheiner E., Marth E., Buzina W. (2009). In-vivo *Candida* biofilms in scanning electron microscopy. Med. Mycol..

[77] Nett J., Lincoln L., Marchillo K., Andes D. (2007). Beta-1,3 glucan as a test for central venous catheter biofilm infection. J. Infect. Dis..

[78] Seneviratne C.J., Wang Y., Jin L., Abiko Y., Samaranayake L.P. (2010). Proteomics of drug resistance in *Candida glabrata* biofilms. Proteomics.

[79] Kuhn D.M., Chandra J., Mukherjee P.K., Ghannoum M.A. (2002). Comparison of biofilms formed by *Candida albicans* and *Candida parapsilosis* on bioprosthetic surfaces. Infect. Immun..

[80] Kucharikova S., Tournu H., Lagrou K., Van Dijck P., Bujdakova H. (2011). Detailed comparison of *Candida albicans* and *Candida glabrata* biofilms under different conditions and their susceptibility to caspofungin and anidulafungin. J. Med. Microbiol..

[81] Riera M., Mogensen E., d’Enfert C., Janbon G. (2012). New regulators of biofilm development in *Candida glabrata*. Res. Microbiol..

[82] Juarez-Cepeda J., Orta-Zavalza E., Canas-Villamar I., Arreola-Gomez J., Perez-Cornejo G.P., Hernandez-Carballo C., Gutierrez-Escobedo G., Castaño I., De Las Peñas A. (2015). The *EPA2* adhesin encoding gene is responsive to oxidative stress in the opportunistic fungal pathogen *Candida glabrata*. Curr. Genet..

[83] Enkler L., Richer D., Marchand A.L., Ferrandon D., Jossinet F. (2016). Genome engineering in the yeast pathogen *Candida glabrata* using the CRISPR-Cas9 system. Sci. Rep..

[84] Cen Y., Timmermans B., Souffriau B., Thevelein J.M., Van Dijck P. (2017). Comparison of genome engineering using the CRISPR-Cas9 system in *C. glabrata* wild-type and *lig4* strains. Fungal Genet. Biol..

